# Perceived Social Support and Associated Factors Among Community-Dwelling Older Adults With Frailty and Pre-frailty in Hangzhou, China

**DOI:** 10.3389/fpsyt.2022.944293

**Published:** 2022-07-14

**Authors:** Juan Fang, Jianping Ren, Lixian Ren, Xiantao Qiu, Shuang Yuan, Wenting Wang, Jinjing Wang

**Affiliations:** Department of Health Management, School of Public Health, Hangzhou Normal University, Hangzhou, China

**Keywords:** aged, frailty, pre-frailty, social, support

## Abstract

**Objectives:**

The present study aimed to explore the social support among community-dwelling older adults with frailty and pre-frailty and to ascertain associated factors.

**Methods:**

The frailty status of the participant was assessed *via* the Chinese FRAIL Scale. The dependent variable, level of social support, was evaluated using the Social Support Rating Scale (range: 12–66). This study explored the influencing factors from three aspects containing sociodemographic characteristics, family environment, and community environment. Independent-sample *t*-test, Analysis of Variance, and multiple linear regression analyses were conducted to examine determinants of social support.

**Results:**

There were significant differences in overall social support between non-frail, pre-frail, and frail participants [38.01 (SD = 6.48) vs. 33.62 (SD = 6.25) vs. 30.50 (SD = 6.68), *F* = 62.157, *p* < 0.001]. Older adults with frailty and pre-frailty who were single would have lower levels of overall social support. In the pre-frail group, living alone was associated with lower overall social support. In contrast, the relationship with children and the availability of recreational activities were associated factors for the frail group.

**Conclusions:**

The level of social support among frail and pre-frail community-dwelling older adults was lower than the robust older adults and influenced by different factors according to the frailty category, which suggests taking targeted measures for social support improvement.

## Introduction

Frailty, a significant public health issue (1), is a multisystem age-related syndrome with an individual's increased vulnerability to adverse health outcomes when exposed to stressors due to declined physiological reserve (2–4). Pre-frailty is a dynamic syndrome, a transitional and potentially reversible risk state before the onset of frailty, including four subtypes: physical, social, cognitive, and nutritional (5). Physical frailty, described as pre-disability (6), is mainly characterized by three or more of five components: weakness, slowness, shrinking, exhaustion, and low physical activity, according to Fried et al. (2). Physical pre-frailty is linked with alterations in physiology and pathophysiology (5) and is also commonly identified by the Fried Frailty Phenotype with one or two of the above components (2). Community-dwelling older adults are prone to developing frailty, and the incidence of frailty was significantly higher in pre-frail individuals than in robust individuals (7). Frail and pre-frail older adults frequently use primary and hospital care (8). Improving prognosis and preventing deterioration from a pre-frail to frail status is vital to promote healthier aging and reduce the burden on health systems (9).

Social support is the perception and actuality that one is cared for by others, which is meaningful and will provide care for frail people (10). Previous studies have demonstrated associations between social support and frailty (11–13). A study from the USA found that, for older adults who were already frail, increased social support was related to less-steep increases in frailty (13). Social isolation accompanying aging accelerates frailty and worsens chronic health issues (14). Lack of social support is one of the various pathophysiologic mechanisms for the development of frailty (12). Several underlying pathways of social support on frailty have been explored (15, 16). One is the physiological pathway, in which social support could prevent the worsening of frailty by reducing the disease burden. Another study elucidated the psychological and behavioral health pathway (16). On one hand, social support could delay the deterioration of frailty by decreasing depressive symptoms (16). On the other hand, older adults with higher-level social support may be more motivated to participate in physical activity (17), which is a cornerstone for preventing and even reversing frailty (16, 18). Correspondingly, according to the socio-ecological theory, we speculated that many older persons might be frailer without enough individual-, family- and community-level support.

Social support is a protective factor for both frailty and pre-frailty (19), which was also elucidated in a previous study conducted in China (16). All persons with frailty should receive social support to address unmet needs and encourage adherence to a Comprehensive Management Plan (6). Given that there has been a worldwide increase in the frail population (20–22), it is necessary to explore possible associated factors related to social support in older adults with frailty, and it is urgent to establish and implement social support strategies among older people. Based on previous studies (23, 24), social support was associated with older adults' sociodemographic characteristics, including city, age, living conditions, marital status, and self-rated health. In the present research, we hypothesize it would also be related to family and community environments, which have been underexplored. The purpose of this study was to explore such associations among the community-dwelling older adults in Hangzhou of Zhejiang Province, China, and to provide scientific evidence for policymakers to improve the level of social support for older people with frailty and pre-frailty.

## Methods

### Participants and Procedure

A community-based cross-sectional survey was undertaken from July 2021 to September 2021 in Hangzhou City, Zhejiang Province, China. Participants were recruited *via* the method of multi-stage typical sampling. Firstly, three administrative districts (Xihu District, Gongshu District, Shangcheng District) were selected among 10 districts in Hangzhou according to geographical locations and economic development. Secondly, three community healthcare centers of sub-districts were chosen from each district, with nine community healthcare centers chosen eventually.

All adults aged 60 years and over who were in the community healthcare center and could communicate in Chinese were invited to participate in the investigation. Respondents were excluded if they met one of the following criteria: (1) with cognitive impairment; (2) with hearing or visual impairment that might hinder communication; (3) with aphasia; or (4) being unwilling to complete the investigation owing to various reasons.

The sample size was estimated by the calculation formula of the single sample mean, with social support assessed by the same measure was 32.76 ± 6.06 among older adults in Anhui Province (10), with a margin of error of 1. The rejection rate of 30% was also considered. We aimed to recruit a minimum of 202 participants. Finally, 600 older adults who visited the community healthcare centers were invited to participate, and 57 declined. Hence, 543 older adults were investigated face-to-face by four trained investigators. During the survey, investigators highlighted the nature of the study and ensured the anonymity of data collection. For the participants who completed the questionnaire by themselves, investigators explained the use of the questionnaire and answered questions about the ambiguous items if necessary. For the others, researchers filled in questionnaires based on their responses. There were no missing data due to investigators checking questionnaires on the spot.

### Measures

#### Frailty

The frailty status of the older adults was assessed *via* the FRAIL (Fatigue, Resistance, Ambulation, Illnesses, and Loss of weight) Scale, which contains five self-report indicators with dichotomous responses: fatigue, resistance, ambulation, illness, and loss of weight (25). It is a purely self-report physical dimensional scale and has been culturally adapted and validated in China (26). The scores range from 0 to 5, with a score of 0 representing robust, 1–2 for pre-frail, and 3–5 for frail in the original version (25), while a score of 1 representing pre-frailty and 2–5 for frailty in Chinese version (26). The Chinese FRAIL scale had high reliability and validity in Chinese community-dwelling older adults (26).

#### Social Support

Social support was measured by the Social Support Rating Scale (SSRS) (27), which was developed and used mostly in China with excellent reliability and validity among the older adults (28). It contains ten items with three dimensions: subjective support (4 items and ranges from 8 to 32), objective support (3 items and ranges from 1 to 22), and support utilization (3 items and ranges from 3 to 12). The scale's total score ranges from 12 to 66, with higher scores signifying greater social support. Subjective support refers to an individual's emotional experience and satisfaction degree of being respected, supported, and understood in society. Objective support refers to visible or practical support, including direct material assistance and the presence and participation of social networks and group relationships. Support utilization refers to the degree of involvement in social activities, as well as the frequency and extent of seeking social support when encountering adverse events.

#### Sociodemographic Characteristics

The sociodemographic characteristics included gender, age, marital status (married/single), educational level (uneducated/ elementary school/ junior high school/senior high school/college or above), monthly income [Q1 (<$300), Q2 ($ 300–600), Q3 ($ 601–1051), Q4 (≥$1052)].

#### The Environment of Family and Community

The factors regarding the family situation are as follows: the number of children ( ≤1/≥2), living alone (yes/no), financial support from children (yes/no), moral support from children (yes/no), relationship with children (poor/fair/good), company of children (rarely/sometimes/often).

The second part is about the community environment, containing medical institutions accessible in 15 min (yes/no), sports fields accessible in 15 min (yes/no), nursing homes accessible in 15 min (yes/no), the management of community (bad/fair/good), availability of recreational activities (sports, music, arts, et al.) and health education (yes/no).

All the measures for sociodemographic characteristics, the environment of family, and the environment of the community were selected and modified according to a previous study in China (29) and expert consultation. A pilot test with 30 older adults—half male, half female- at one community healthcare center by convenience sampling in June 2021, including 10 in the age group of 60–69, 10 in the age group of 70–79, and 10 in the age group of 80–89. Some adjustments were made to respondents' unclear items during the pilot test.

### Ethical Considerations

This study was approved by the Ethics Committee of the School of Public Health, Hangzhou Normal University (Number: 20210012). All the participants signed an informed consent statement before participation and were notified that they were free to accept or reject the invitation to participate in the investigation.

### Data Analysis

This study used the Statistical Package for the Social Sciences version 22.0 for data analysis. Independent-samples *t*-test or Analysis of Variance (ANOVA) was performed to preliminarily examine the associations between study variables and social support, including three dimensions and the overall social support. To further identify contributing factors of social support, multiple linear regression analyses with social support regarded as the dependent variable and statistically significant variables as independent ones were performed according to the frailty category (non-frailty, pre-frailty, and frailty). The significance level for all analyses was set at 0.05, two-tailed.

## Results

### Sociodemographic Characteristics

In this study, 239 (44.0%) were non-frail, 169 (31.1%) were pre-frail, and 135 (24.9%) were frail among 543 respondents using the cut-point of the Chinese FRAIL Scale, and more than half (58.6%) were female. Their age ranged from 60 to 94 years, with an average of (70.99 ± 8.26) years. 21.9% of the participants were single (unmarried, divorced, or widowed). Only 15.8% of participants had not been educated. More than three-quarters (76.3%) of the participants have a monthly income ranging from $300 to $1,051. The detailed sociodemographic characteristics are shown in Table 1.

**Table 1:** Demographic characteristics of community-dwelling older adults.

**Variable**	**Total**		**Frailty category**	
	**(*N* = 543)**	**Non-frail (*N* = 239)**	**Pre-frail (*N* = 169)**	**Frail (*N* = 135)**
**Gender**
Male	225 (41.4)	100 (41.8)	72 (42.6)	53 (39.3)
Female	318 (58.6)	139 (58.2)	97 (57.4)	82 (60.7)
**Age (years)**
60–69	259 (47.7)	155 (64.9)	74 (43.8)	30 (22.2)
70–79	185 (34.1)	71 (29.7)	62 (36.7)	52 (38.5)
≥80	99 (18.2)	13 (5.4)	33 (19.5)	53 (39.3)
**Marital status**
Married	424 (78.1)	203 (84.9)	139 (82.2)	82 (60.7)
Single^*^	119 (21.9)	36 (15.1)	30 (17.8)	53 (39.3)
**Educational level**
Uneducated	86 (15.8)	36 (15.1)	16 (9.5)	34 (25.2)
Elementary school	164 (30.2)	63 (26.4)	58 (34.3)	43 (31.9)
Junior high school	146 (26.9)	73 (30.5)	45 (26.6)	28 (20.7)
Senior high school	90 (16.6)	40 (16.7)	31 (18.3)	19 (14.1)
College or above	57 (10.5)	27 (11.3)	19 (11.2)	11 (8.1)
**Monthly income ($)**
<300	90 (16.6)	50 (20.9)	23 (13.6)	17 (12.6)
300–600	212 (39.0)	99 (41.4)	53 (31.4)	60 (44.4)
601–1,051	189 (34.8)	70 (29.3)	71 (42.0)	48 (35.6)
≥1,052	52 (9.6)	20 (8.4)	22 (13.0)	10 (7.4)

* *Single: including unmarried, divorced, or widowed*.

### Social Support

As shown in Table 2, the mean SSRS score for all respondents was 34.78 (SD = 7.15, range = 12–66). Specifically, the overall score of SSRS for non-frail respondents was 38.01 ± 6.48, with the objective support 9.39 ± 2.47, subjective support 20.93 ± 4.06, support utilization 7.69 ± 2.26; for pre-frail ones was 33.62 ± 6.25 (objective support 8.35 ± 2.17, subjective support 19.18 ± 3.94, support utilization 6.09 ± 1.93); and for frail ones, the total score of this scale was 30.50 ± 6.68 (objective support 7.54 ± 2.40, subjective support 17.58 ± 4.15, support utilization 5.39 ± 1.70). There was a statistically significant difference in social support between different frailty statuses (*F* = 62.157, *p* < 0.001).

**Table 2:** Social support of community-dwelling older adults according to the frailty category.

**Dimension**	**Total (*N* =543)**	**Non-frail (*N* =239)**	**Pre-frail (*N* =169)**	**Frail (*N* =135)**	***F***	***P***
Objective support	8.61 ± 2.48	9.39 ± 2.47	8.35 ± 2.17	7.54 ± 2.40	27.828	<0.001
Subjective support	19.55 ± 4.26	20.93 ± 4.06	19.18 ± 3.94	17.58 ± 4.15	30.608	<0.001
Support utilization	6.62 ± 2.26	7.69 ± 2.26	6.09 ± 1.93	5.39 ± 1.70	63.622	<0.001
Overall	34.78 ± 7.15	38.01 ± 6.48	33.62 ± 6.25	30.50 ± 6.68	62.157	<0.001

### Factors Associated With Social Support: Results of Bivariate Analysis

As shown in Table 3, *t*-tests or ANOVA showed that the total social support score differed by age and marital status for all frailty categories (*p* < 0.05). We also found a statistically significant association between social support and educational level for both robust participants and older adults with frailty (*p* < 0.05). Additionally, monthly income was the potential factor only for non-frail older persons (*p* = 0.006). The influence of demographic characteristics on objective support, subjective support, and support utilization according to the frailty category was shown in Supplementary Table 1. In the non-frail group, age and educational level were associated with three dimensions; marital status was associated with objective and subjective support; monthly income was associated with objective support and support utilization. Among the pre-frail participants, marital status was an associated factor for objective support; marital status and monthly income were for subjective support; gender and age were for support utilization. For frail older adults, age was associated with subjective support and support utilization; marital status was associated with objective and subjective support; the educational level was associated with subjective support and support utilization.

**Table 3:** The impact of demographic characteristics on social support according to the frailty category.

**Variable**	**Non-frail**		**Pre-frail**		**Frail**	
	**Social support (*N* =239)**	***P***	**Social support (*N* =169)**	***P***	**Social support (*N* =135)**	***P***
Gender		0.321		0.337		0.266
Male	38.50 ± 6.53		33.08 ± 6.15		31.30 ± 7.32	
Female	37.65 ± 6.45		34.02 ± 6.33		29.99 ± 6.22	
Age (years)		0.000		0.047		0.011
60–69	39.12 ± 6.03		34.95 ± 5.94		32.70 ± 5.90	
70–79	35.20 ± 6.06		32.79 ± 5.79		31.29 ± 6.67	
≥80	40.08 ± 9.16		32.21 ± 7.30		28.49 ± 6.65	
Marital status		0.000		0.000		0.000
Married	39.00 ± 6.03		34.81 ± 5.55		33.15 ± 5.92	
Single^*^	32.39 ± 6.13		28.10 ± 6.46		26.42 ± 5.67	
Educational level		0.000		0.195		0.011
Uneducated	33.86 ± 5.79		32.06 ± 7.76		29.38 ± 5.63	
Elementary school	36.70 ± 6.46		33.86 ± 5.93		29.05 ± 7.07	
Junior high school	38.44 ± 5.93		33.42 ± 5.90		31.54 ± 6.32	
Senior high school	39.83 ± 5.93		32.52 ± 6.88		30.79 ± 7.29	
College or above	42.74 ± 5.73		36.47 ± 5.09		36.55 ± 4.74	
Monthly income ($)		0.006		0.054		0.133
<300	36.36 ± 5.32		35.17 ± 6.21		30.53 ± 4.24	
300–600	37.71 ± 6.42		32.32 ± 6.30		29.47 ± 6.79	
601–1,051	38.40 ± 7.05		33.30 ± 6.10		30.92 ± 7.28	
≥1,052	42.25 ± 5.71		36.18 ± 5.97		34.70 ± 5.01	

* *Single: including unmarried, divorced, or widowed*.

In terms of family environment (Table 4), living alone or not (*p* = 0.001), and relationship with children (*p* < 0.05) might be the impact factors of social support for three groups. Besides, getting moral support from children or not would have significant differences in social support perceived by pre-frail and frail older adults in the community (*p* < 0.05). For non-frail ones, the number of children was also associated with the level of social support (*p* = 0.000). The impact of family environment on objective support, subjective support, and support utilization (see Supplementary Table 2) according to the frailty category were also explored. In the non-frail group, the number of children, living alone or not, and relationship with children were associated with objective and subjective support. In addition, getting moral support from children or not would be an associated factor for subjective support. In the pre-frail group, living alone or not and getting financial support from children or not were associated with objective support and support utilization, respectively. The associated factors for subjective were living alone or not, getting moral support from children or not, relationship with children, and children's company. For frail respondents, relationship with children was an associated factor for three dimensions; living alone or not and getting moral support from children or not were potential factors for objective and subjective support; children's company was only an impact factor for objective support.

**Table 4:** The impact of family environment on social support according to the frailty category.

**Variable**	**Non-frail**		**Pre-frail**		**Frail**	
	**Social support (*N* =239)**	***P***	**Social support (*N* =169)**	***P***	**Social support (*N* =135)**	***P***
The number of children		0.000		0.678		0.527
≤1	39.61 ± 6.24		33.41 ± 6.19		30.98 ± 7.39	
≥2	36.45 ± 6.35		33.82 ± 6.33		30.22 ± 6.24	
Living alone		0.000		0.000		0.000
No	38.49 ± 6.17		34.43 ± 5.56		31.84 ± 6.22	
Yes	31.25 ± 7.09		25.88 ± 7.32		25.62 ± 6.06	
Financial support from children		0.902		0.102		0.297
No	37.98 ± 6.35		33.20 ± 6.13		30.25 ± 6.80	
Yes	38.12 ± 7.15		35.11 ± 6.54		31.90 ± 5.91	
Moral support from children		0.167		0.007		0.001
No	32.00 ± 7.35		31.72 ± 5.57		28.54 ± 6.81	
Yes	35.94 ± 6.11		34.49 ± 6.38		32.22 ± 6.10	
Relationship with children		0.000		0.017		0.000
Poor	30.57 ± 6.19		28.60 ± 9.86		22.55 ± 7.05	
Fair	33.15 ± 6.13		31.21 ± 6.55		28.08 ± 5.43	
Good	38.90 ± 6.10		34.21 ± 5.92		31.96 ± 6.15	
Children's company		0.861		0.101		0.116
Rarely	37.75 ± 7.33		31.52 ± 6.87		28.10 ± 8.27	
Sometime	37.60 ± 7.44		32.57 ± 6.44		29.19 ± 6.68	
Often	38.17 ± 6.02		34.23 ± 6.02		31.20 ± 6.24	

The results of analyses in the aspect of community situation in Table 5 indicated that the level of social support might be related to community management for pre-frail respondents (*p* = 0.004). Differently, for frail ones, accessibility to medical institutions within 15 min (*p* = 0.008) and utilization of recreational activities (*p* = 0.001) might be associated factors for perceived social support. Supplementary materials also present the association between the community environment and three dimensions (see Supplementary Table 3). For non-frail older adults, community management might be related to objective support; accessibility to medical institutions, sports fields, and nursing homes within 15 min might be related to subjective support; availability of health education was a potential factor for support utilization. Community management was the only associated factor for subjective support for pre-frail older adults. In the frail group, the availability of recreational activity was related to three dimensions. Community management was associated with objective support. Accessibility to medical institutions and nursing homes within 15 min would significantly differ in subjective support.

**Table 5:** The impact of community environment on social support according to the frailty category.

**Variable**	**Non-frail**		**Pre-frail**		**Frail**	
	**Social support (*N* =239)**	***P***	**Social support (*N* =169)**	***P***	**Social support (*N* =135)**	***P***
Medical institutions accessible		0.180		0.296		0.008
No	36.94 ± 6.51		32.32 ± 7.61		28.32 ± 6.91	
Yes	38.30 ± 6.46		33.82 ± 6.03		31.56 ± 6.33	
Sports fields accessible		0.096		0.523		0.070
No	36.78 ± 6.60		34.24 ± 7.03		28.78 ± 6.46	
Yes	38.40 ± 6.41		33.47 ± 6.06		31.13 ± 6.68	
Nursing homes accessible		0.175		0.638		0.067
No	37.08 ± 6.21		33.27 ± 6.65		29.06 ± 6.58	
Yes	38.36 ± 6.56		33.77 ± 6.11		31.27 ± 6.64	
Management of community		0.462		0.004		0.365
Bad	36.53 ± 7.54		35.21 ± 5.37		28.33 ± 6.68	
Fair	38.78 ± 6.27		31.81 ± 6.51		30.26 ± 6.41	
Good	37.91 ± 6.46		35.00 ± 5.77		31.22 ± 6.96	
Availability of recreational activity		0.587		0.705		0.001
No	37.88 ± 6.17		33.53 ± 6.26		29.65 ± 6.47	
Yes	38.56 ± 7.74		34.00 ± 6.30		34.46 ± 6.31	
Availability of health education		0.059		0.221		0.149
No	37.71 ± 6.39		33.27 ± 6.12		30.12 ± 6.60	
Yes	40.14 ± 6.82		34.60 ± 6.55		32.29 ± 6.86	

### Factors Associated With Social Support: Results of Multivariate Analysis

For robust older adults, compared with the 60–69 years age group, participants in the 70–79 years age group reported a lower level of social support (β = −0.151, *p* = 0.011), objective support (β = −0.227, *p* = 0.000), and subjective support (β = −0.138, *p* = 0.022), while participants in the ≥80 years age group reported higher level of social support (β = 0.157, *p* = 0.008) and support utilization (β = 0.177, *p* = 0.007). Single ones, including unmarried, divorced, and widowed ones, tend to report lower scores in social support (β = −0.202, *p* = 0.001) and subjective support (β = −0.253, *p* = 0.000). It was found that illiterate participants perceived lower scores on social support than those who were educated. Compared with the uneducated respondents, respondents with educational level of junior high school and senior high school reported higher scores in subjective support (β = 0.234, *p* = 0.005; β = 0.245, *p* = 0.001), and respondents with educational level of college or above reported higher scores in three dimensions (β = 0.330, *p* = 0.000; β = 0.174, *p* = 0.015; β = 0.268, *p* = 0.010). The older adults who lived alone perceived lower level of objective support (β = −0.233, *p* = 0.000) than those who did not live alone. Older adults with one or no children had higher social support scores than those with two or more children (β = −0.149, *p* = 0.021). Respondents with poor relationships with children were more prone to poor social support (β = 0.368, *p* = 0.001) and subjective support (β = 0.331, *p* = 0.005). Among the three dimensions, the number of potential associated factors on support utilization was the least. Older adults who lived in the community with fair (β = 0.235, *p* = 0.025) and good (β = 0.234, *p* = 0.028) management tend to report higher scores in objective support than those lived in community with bad management. All results are displayed in Supplementary Table 4.

The associated factors of social support among pre-frail and frail participants are presented in Supplementary Tables 5, 6. Lower perceived social support level was observed among both the aged pre-frail and frail people who were single (β = −0.289, *p* = 0.000; β = −0.292, *p* = 0.002). Specifically, single pre-frail older adults reported lower scores in objective and subjective support than married ones (β = −0.295, *p* = 0.000; β = −0.314, *p* = 0.000), so as the frail ones (β = −0.302, *p* = 0.000; β = −0.232, *p* = 0.022). Living alone negatively affected social support (β = −0.203, *p* = 0.012) among pre-frail older adults and objective support (β = −0.388, *p* = 0.000; β = −0.310, *p* = 0.000) in pre-frail and frail group. Frail participants, who were in fair and good relationships with children were more likely to perceive a higher level of social support (β = 0.240, *p* = 0.042; β = 0. 383, *p* = 0.003), objective support (β = 0.314, *p* = 0.006; β = 0. 315, *p* = 0.012), and subjective support (β = 0. 143, p = 0.248; β = 0. 317, *p* = 0.019). Frail older adults who were offered opportunities to participate in recreational activities showed greater social support, objective support, and support utilization than those who had no opportunities (β = 0.197, *p* = 0.014; β = 0.156, *p* = 0.015; β = 0.384, *p* = 0.000).

In addition, in the pre-frail group, older adults with a monthly income of $300-600 reported lower scores in subjective support than those with < $300 (β = −0.239, *p* = 0.024). Participants who were often companied with adult children reported a higher level of subjective support (β = 0.208, *p* = 0.032). Getting financial support from children positively affected support utilization (β = 0.238, *p* = 0.002). In the frail group, the educational level of college or above and living in a community with good management positively influenced subjective support (β = 0.199, *p* = 0.029) and objective support (β = 0.259, *p* = 0.025), respectively. For the pre-frail older persons, the factors affecting subjective support were the most, but for the frail older persons, the factors affecting objective support were the most.

## Discussion

The level of social support (34.78 ± 7.15) among participants in this study was similar to the social support level of community-dwelling older adults in Fuzhou, Fujian Province (34.99 ± 5.94) (23), as well as the results of a meta-analysis about social support among Chinese older adults aged 60 years (34.047) (30). For pre-frail and frail older adults, our results were nearly consistent with a previous study conducted in Anhui Province, which reported that the total social support score was 33.43 ± 5.80 and 30.96 ± 5.99 among older adults with pre-frailty in 60–76 years and ≥77 years age group, respectively (10). Furthermore, the total score was 31.57 ± 6.56 and 29.06 ± 6.32 among older adults with frailty in both age groups (10). Though the level of social support among the older adults in Zhejiang Province and Anhui Province was similar, this should be presented cautiously, as the frailty status was assessed by the FRAIL Scale and Frailty Index (10), respectively. Additionally, we recruited the target participants from the community healthcare centers, and they selected the individuals from rural villages (10). Importantly, the more severe the frailty, the lower the level of social support. Interventions should be taken to enhance social support as early as possible, especially for pre-frail persons.

We found the same results as previous studies (23) that the older adults who were unmarried, divorced, and widowed might perceive less social support, mainly subjective and objective support, than those who had spouses. Spouses might offer physical and emotional companionship. With a healthier marital status, older people may be prone to perceive more available social support, and they may enlarger their social networks more easily (31). Therefore, frailty management, especially social support, is called for older pre-frail and frail adults who are unmarried, divorced, and widowed. The effect of marital status on social support among the older adults is not uniformly positive, neutral, or negative (32). Hence, more longitudinal studies are needed. The present study also showed that monthly income was only associated with subjective support among pre-frail participants. Surprisingly, older adults with a monthly income of $300–600, compared with older adults having a monthly income of <$300, perceived less subjective support in our investigation. This phenomenon could be because the older adults with a monthly income of $300–600 might have more subjective needs for social support than those with lower monthly incomes. In addition, the linear regression showed that the educational level of college or above actively impacted subjective support among the frail older adults. Education is beneficial for older adults to acquire better socioeconomic status and job that determines wider social network and more social resources (30). Thus, the illiterate frail older adults in the community should be the target of the intervention programs on social support, especially subjective support.

There were also some factors regarding the family of the older adults contributing to the level of social support. Firstly, it was indicated that pre-frail and frail responders living alone might tend to perceive less social support, especially objective support, which is consistent with the previous results (33). A possible explanation might be that those living alone need more support with, for example, household chores, local transportation, or someone to talk to about their worries and ask for advice (33). Moreover, older adults living alone reported poorer health, more comorbid medical conditions, and more physical limitations, which increases the need for caregivers (33). Cohabiting with adult children could provide greater financial, instrumental, emotional, and physical support (34). Notably, some single (unmarried, divorced, and widowed) respondents lived alone, so the results should be presented cautiously. Secondly, the quality of relationships with adult children was positively associated with overall social support, objective support, and subjective support perceived by the frail older adults. The quality of relationships with children played an important for elders, especially for widowhood ones (34). A stable family may be one reason for a higher objective support score (35). The fact that 89.1% of older adults perceived their children as the primary source of subjective support might explain the above phenomenon partly (23). Besides, the fact in the previous could also explain why the more children accompanied, the higher the score of subjective support among the pre-frail older persons (23). Finally, the pre-frail older adults who had received financial support from adult children, compared to those who had not, perceived higher scores in support utilization. One reason might be that they were more confident and active in seeking social support through various social networks. It might also be due to the significance of intergenerational financial support to subjective wellbeing among older adults (36).

Furthermore, the frequent opportunities provided by the community to participate in recreational activities, such as singing, dancing, painting, and walking, positively impact overall social support, objective support, and support utilization among frail older adults. There are two possible reasons for this result. One is that recreational activity can enhance social confidence, communication, reciprocal relationship, and other interpersonal skills (37). Besides socialization benefits, recreational activities are encouraged for older people as they could increase their smiles and raise their joy (38). The results also indicated that the great management of the community was a positive factor for objective support among frail older adults. The reason perhaps was that a well-managed community is more likely to provide an excellent physical and social environment. It was recognized that a community should be dynamic to support changes in the older citizenry (39), and a community's perceived age-friendliness is associated with the quality of life among older adults (40).

It is worth mentioning that social support decreased with aging in the older persons (24), which was only found in our study among the robust older persons. It was indicated that the association between social support and frailty was negative in the ≥77 years age group, compared to the 60–76 years age group among the Chinese (10). The relationships between age, social support, and progression of frailty need further research on mediating effects and more longitudinal study. An interesting phenomenon that the number of children only works in the robust population but not in the pre-frail and frail population needs further exploration to understand the impact factors and the interaction better.

To our knowledge, this study is the first to explore the comprehensive associated factors of social support among frail and pre-frail older adults in Chinese community centers. However, some limitations should be acknowledged. First, given the cross-sectional research design of our study, causality cannot be ascertained. Second, the scarcity of previous studies aimed at a comprehensive understanding of the contributing factors for social support among community-dwelling older adults with pre-frailty and frailty limited the comparability of our findings. Third, the data were only collected from Hangzhou City, China, limiting the findings' generalization to other regions.

## Conclusion

The level of social support among the frail and pre-frail older adults in Hangzhou, China, needs improvement. Older adults affected with frailty and pre-frailty may present different levels of social support based on sociodemographic characteristics, family environment, and the community. Social support improvement should be integrated into frailty management.

## Data Availability Statement

The raw data supporting the conclusions of this article will be made available by the authors, without undue reservation.

## Ethics Statement

The studies involving human participants were reviewed and approved by the Ethics Committee of the School of Public Health, Hangzhou Normal University (Number: 20210012). The patients/participants provided their written informed consent to participate in this study.

## Author Contributions

JF, JR, and LR conceived and designed the study. JF, LR, XQ, and SY participated in the acquisition of data. JF and LR analyzed the data and drafted the manuscript. JR and WW gave advice on methodology. JR and JW revised the manuscript. All authors read and approved the final manuscript.

## Funding

This work was supported by the National Natural Science Foundation of China (71874047) and the Zhejiang Province Public Welfare Technology Application Research Project (LGF21G030003).

## Conflict of Interest

The authors declare that the research was conducted in the absence of any commercial or financial relationships that could be construed as a potential conflict of interest.

## Publisher's Note

All claims expressed in this article are solely those of the authors and do not necessarily represent those of their affiliated organizations, or those of the publisher, the editors and the reviewers. Any product that may be evaluated in this article, or claim that may be made by its manufacturer, is not guaranteed or endorsed by the publisher.
