# Supplementary material for: Perceived Social Support and Associated Factors Among Community-Dwelling Older Adults With Frailty and Pre-frailty in Hangzhou, China
Source: Front Psychiatry. 2022 Jul 14;13:944293. doi: 10.3389/fpsyt.2022.944293 (PMC9329702; doi:10.3389/fpsyt.2022.944293)
Supplement: Supplementary file 1 [file Data_Sheet_1.doc]

**Supplementary Material**

**TABLE 1 |** The impact of demographic characteristics on three dimensions of social support according to the frailty category.

| **Variable** | **Non-frail (*N* =239)** | | | | | | **Pre-frail (*N* =169)** | | | | | | **Frail (*N* =135)** | | | | | |
| --- | --- | --- | --- | --- | --- | --- | --- | --- | --- | --- | --- | --- | --- | --- | --- | --- | --- | --- |
|  | **Objective support** | ***P*** | **Subjective support** | ***P*** | **Support utilization** | ***P*** | **Objective support** | ***P*** | **Subjective support** | ***P*** | **Support utilization** | ***P*** | **Objective support** | ***P*** | **Subjective support** | ***P*** | **Support utilization** | ***P*** |
| Gender |  | 0.079 |  | 0.286 |  | 0.325 |  | 0.528 |  | 0.370 |  | 0.046 |  | 0.117 |  | 0.184 |  | 0.281 |
| Male | 9.72±2.60 |  | 21.26±3.84 |  | 7.52±2.31 |  | 8.47±2.13 |  | 18.86±3.69 |  | 5.75±1.64 |  | 7.94±2.78 |  | 18.17±4.30 |  | 5.19±1.36 |  |
| Female | 9.15±2.35 |  | 20.69±4.22 |  | 7.81±2.23 |  | 8.26±2.21 |  | 19.41±4.12 |  | 6.35±2.10 |  | 7.28±2.10 |  | 17.20±4.04 |  | 5.51±1.88 |  |
| Age (years) |  | 0.000 |  | 0.000 |  | 0.020 |  | 0.099 |  | 0.282 |  | 0.031 |  | 0.061 |  | 0.002 |  | 0.002 |
| 60-69 | 9.89±2.47 |  | 21.66±3.55 |  | 7.57±2.23 |  | 8.74±2.18 |  | 19.68±3.57 |  | 6.53±2.15 |  | 8.07±2.23 |  | 19.37±3.70 |  | 19.37±3.70 |  |
| 70-79 | 8.25±2.03 |  | 19.31±4.20 |  | 7.63±2.28 |  | 8.13±1.85 |  | 18.98±3.88 |  | 5.68±1.51 |  | 7.85±2.20 |  | 17.96±4.05 |  | 17.96±4.05 |  |
| ≥80 | 9.62±2.72 |  | 21.08±6.34 |  | 9.38±2.02 |  | 7.88±2.61 |  | 18.42±4.72 |  | 5.91±1.99 |  | 6.94±2.59 |  | 16.19±4.10 |  | 16.19±4.10 |  |
| Marital status |  | 0.000 |  | 0.000 |  | 0.991 |  | 0.000 |  | 0.000 |  | 0.987 |  | 0.000 |  | 0.000 |  | 0.648 |
| Married | 9.72±2.37 |  | 21.60±3.69 |  | 7.69±2.28 |  | 8.86±1.71 |  | 19.86±3.65 |  | 6.09±1.86 |  | 8.65±1.86 |  | 19.06±3.98 |  | 5.44±1.54 |  |
| Single^*^ | 7.53±2.21 |  | 17.17±4.09 |  | 7.69±2.19 |  | 6.00±2.56 |  | 16.00±3.72 |  | 6.10±2.29 |  | 5.83±2.13 |  | 15.28±3.30 |  | 5.30±1.93 |  |
| Educational level |  | 0.000 |  | 0.000 |  | 0.009 |  | 0.459 |  | 0.207 |  | 0.731 |  | 0.063 |  | 0.002 |  | 0.002 |
| Uneducated | 8.47±1.86 |  | 18.33±3.74 |  | 7.06±2.07 |  | 8.19±2.93 |  | 18.13±4.30 |  | 5.75±1.81 |  | 7.41±1.84 |  | 16.29±3.77 |  | 16.29±3.77 |  |
| Elementary school | 8.84±2.07 |  | 20.03±4.31 |  | 7.83±2.41 |  | 8.38±1.75 |  | 19.24±4.02 |  | 6.24±2.30 |  | 7.12±2.38 |  | 16.74±4.20 |  | 16.74±4.20 |  |
| Junior high school | 9.47±2.62 |  | 21.71±4.00 |  | 7.26±2.20 |  | 8.49±2.35 |  | 18.91±3.77 |  | 6.02±1.41 |  | 7.75±2.58 |  | 18.54±4.04 |  | 18.54±4.04 |  |
| Senior high school | 9.45±2.00 |  | 22.28±3.57 |  | 8.10±2.18 |  | 7.81±2.33 |  | 18.84±3.71 |  | 5.87±2.22 |  | 7.32±2.96 |  | 18.1±3.92 |  | 18.16±3.92 |  |
| College or above | 11.59±2.99 |  | 22.37±2.59 |  | 8.78±2.04 |  | 8.95±1.96 |  | 21.05±3.92 |  | 6.47±1.39 |  | 9.45±1.81 |  | 21.36±3.26 |  | 21.36±3.26 |  |
| Monthly income ($) |  | 0.016 |  | 0.057 |  | 0.045 |  | 0.239 |  | 0.031 |  | 0.319 |  | 0.066 |  | 0.140 |  | 0.140 |
| <300 | 9.40±1.93 |  | 19.90±3.25 |  | 7.06±2.17 |  | 9.00±2.39 |  | 19.39±3.35 |  | 6.78±1.93 |  | 8.06±1.20 |  | 17.00±4.02 |  | 17.00±4.02 |  |
| 300-600 | 9.17±2.21 |  | 20.77±4.35 |  | 7.77±2.26 |  | 8.09±1.98 |  | 18.28±3.93 |  | 5.94±2.43 |  | 7.05±2.23 |  | 16.93±4.12 |  | 16.93±4.12 |  |
| 601-1051 | 9.21±2.79 |  | 21.44±4.34 |  | 7.74±2.38 |  | 8.18±2.23 |  | 19.14±3.87 |  | 5.97±1.64 |  | 7.67±2.85 |  | 18.13±4.25 |  | 18.13±4.25 |  |
| ≥1052 | 11.05±3.17 |  | 22.50±2.67 |  | 8.70±1.75 |  | 8.82±2.15 |  | 21.23±4.19 |  | 6.14±1.36 |  | 9.00±1.94 |  | 19.80±3.43 |  | 19.80±3.43 |  |

^*^Single: including unmarried, divorced, or widowed.

**TABLE 2 |** The impact of family environment on three dimensions of social support according to the frailty category.

| **Variable** | **Non-frail (*N* =239)** | | | | | | **Pre-frail (*N* =169)** | | | | | | **Frail (*N* =135)** | | | | | |
| --- | --- | --- | --- | --- | --- | --- | --- | --- | --- | --- | --- | --- | --- | --- | --- | --- | --- | --- |
|  | **Objective support** | ***P*** | **Subjective support** | ***P*** | **Support utilization** | ***P*** | **Objective support** | ***P*** | **Subjective support** | ***P*** | **Support utilization** | ***P*** | **Objective support** | ***P*** | **Subjective support** | ***P*** | **Support utilization** | ***P*** |
| The number of children |  | 0.014 |  | 0.000 |  | 0.091 |  | 0.692 |  | 0.859 |  | 0.592 |  | 0.943 |  | 0.071 |  | 0.070 |
| ≤1 | 9.79±2.67 |  | 21.88±3.80 |  | 7.94±2.14 |  | 8.28±2.10 |  | 19.12±3.76 |  | 6.01±1.96 |  | 7.52±2.79 |  | 18.42±4.32 |  | 5.04±1.35 |  |
| ≥2 | 9.00±2.19 |  | 20.00±4.12 |  | 7.45±2.36 |  | 8.41±2.25 |  | 19.23±4.12 |  | 6.17±1.92 |  | 7.55±2.16 |  | 17.08±3.99 |  | 5.59±1.85 |  |
| Living alone |  | 0.000 |  | 0.001 |  | 0.905 |  | 0.000 |  | 0.000 |  | 0.634 |  | 0.000 |  | 0.000 |  | 0.474 |
| No | 9.63±2.32 |  | 21.17±3.93 |  | 7.63±2.63 |  | 8.73±1.81 |  | 19.58±3.72 |  | 5.88±2.39 |  | 8.28±1.92 |  | 18.23±4.15 |  | 5.33±1.57 |  |
| Yes | 6.06±2.14 |  | 17.56±4.55 |  | 7.70±2.24 |  | 4.69±2.02 |  | 15.31±3.98 |  | 6.12±1.89 |  | 4.83±2.00 |  | 15.21±3.23 |  | 5.59±2.13 |  |
| Financial support from children |  | 0.680 |  | 0.503 |  | 0.688 |  | 0.309 |  | 0.151 |  | 0.000 |  | 0.972 |  | 0.282 |  | 0.127 |
| No | 9.42±2.46 |  | 20.85±3.93 |  | 7.72±2.24 |  | 8.44±2.10 |  | 18.95±3.85 |  | 5.82±1.89 |  | 7.54±2.45 |  | 17.41±4.09 |  | 5.29±1.76 |  |
| Yes | 9.24±2.53 |  | 21.32±4.71 |  | 7.56±2.41 |  | 8.03±2.42 |  | 20.00±4.18 |  | 7.08±1.80 |  | 7.52±2.18 |  | 18.48±4.47 |  | 5.90±1.22 |  |
| Moral support from children |  | 0.087 |  | 0.001 |  | 0.593 |  | 0.305 |  | 0.002 |  | 0.199 |  | 0.000 |  | 0.002 |  | 0.434 |
| No | 8.77±2.11 |  | 19.05±3.92 |  | 7.51±2.34 |  | 8.09±1.98 |  | 17.81±3.57 |  | 5.81±1.47 |  | 6.63±2.47 |  | 16.40±3.87 |  | 5.51±1.94 |  |
| Yes | 9.51±2.52 |  | 21.30±4.00 |  | 7.73±2.25 |  | 8.47±2.26 |  | 19.80±3.95 |  | 6.22±2.11 |  | 8.33±2.04 |  | 18.61±4.14 |  | 5.28±1.46 |  |
| Relationship with children |  | 0.003 |  | 0.000 |  | 0.054 |  | 0.464 |  | 0.009 |  | 0.101 |  | 0.000 |  | 0.000 |  | 0.000 |
| Poor | 7.86±1.35 |  | 17.00±4.80 |  | 5.71±1.98 |  | 7.20±2.95 |  | 15.60±5.18 |  | 5.80±1.92 |  | 4.36±2.66 |  | 13.73±4.15 |  | 13.73±4.15 |  |
| Fair | 8.11±2.24 |  | 17.52±4.45 |  | 7.52±2.17 |  | 8.25±2.51 |  | 17.63±3.98 |  | 5.33±1.43 |  | 6.83±1.83 |  | 15.83±3.40 |  | 15.83±3.40 |  |
| Good | 9.61±2.46 |  | 21.51±3.70 |  | 7.78±2.26 |  | 8.41±2.09 |  | 19.57±3.78 |  | 6.24±1.99 |  | 8.06±2.19 |  | 18.42±3.98 |  | 18.42±3.98 |  |
| Children’s company |  | 0.747 |  | 0.366 |  | 0.156 |  | 0.769 |  | 0.040 |  | 0.441 |  | 0.009 |  | 0.355 |  | 0.355 |
| Rarely | 9.45±2.92 |  | 20.67±4.37 |  | 7.63±2.14 |  | 8.12±2.26 |  | 17.76±4.31 |  | 5.64±1.96 |  | 6.20±3.04 |  | 16.70±4.88 |  | 16.70±4.88 |  |
| Sometime | 9.07±2.52 |  | 20.10±4.52 |  | 8.43±2.13 |  | 8.19±2.52 |  | 18.14±3.50 |  | 6.24±2.02 |  | 7.00±2.16 |  | 16.75±4.02 |  | 16.75±4.02 |  |
| Often | 9.43±2.31 |  | 21.17±3.87 |  | 7.57±2.31 |  | 8.42±2.11 |  | 19.64±3.86 |  | 6.16±1.92 |  | 7.90±2.20 |  | 17.89±4.01 |  | 17.89±4.01 |  |

**TABLE 3 |** The impact of community environment on three dimensions of social support according to the frailty category.

| **Variable** | **Non-frail (*N* =239)** | | | | | | **Pre-frail (*N* =169)** | | | | | | **Frail (*N* =135)** | | | | | |
| --- | --- | --- | --- | --- | --- | --- | --- | --- | --- | --- | --- | --- | --- | --- | --- | --- | --- | --- |
|  | **Objective support** | ***P*** | **Subjective support** | ***P*** | **Support utilization** | ***P*** | **Objective support** | ***P*** | **Subjective support** | ***P*** | **Support utilization** | ***P*** | **Objective support** | ***P*** | **Subjective support** | ***P*** | **Support utilization** | ***P*** |
| Medical institutions accessible |  | 0.334 |  | 0.022 |  | 0.186 |  | 0.808 |  | 0.357 |  | 0.075 |  | 0.228 |  | 0.003 |  | 0.132 |
| No | 9.10±2.35 |  | 19.79±4.07 |  | 8.06±2.24 |  | 8.45±2.72 |  | 18.45±4.07 |  | 5.41±2.13 |  | 7.18±2.56 |  | 16.07±4.22 |  | 5.07±1.74 |  |
| Yes | 9.47±2.50 |  | 21.25±4.02 |  | 7.59±2.27 |  | 8.33±2.09 |  | 19.29±3.92 |  | 6.20±1.89 |  | 7.71±2.31 |  | 18.31±3.94 |  | 5.54±1.66 |  |
| Sports fields accessible |  | 0.209 |  | 0.004 |  | 0.084 |  | 0.652 |  | 0.467 |  | 0.939 |  | 0.398 |  | 0.062 |  | 0.174 |
| No | 9.03±2.41 |  | 19.60±4.16 |  | 8.14±2.24 |  | 8.50±2.44 |  | 19.62±3.81 |  | 6.12±2.24 |  | 7.25±2.05 |  | 16.47±4.31 |  | 5.06±1.76 |  |
| Yes | 9.50±2.48 |  | 21.35±3.95 |  | 7.55±2.26 |  | 8.31±2.11 |  | 19.07±3.97 |  | 6.09±1.86 |  | 7.65±2.52 |  | 17.98±4.04 |  | 5.51±1.67 |  |
| Nursing homes accessible |  | 0.546 |  | 0.012 |  | 0.197 |  | 0.809 |  | 0.709 |  | 0.623 |  | 0.965 |  | 0.018 |  | 0.134 |
| No | 9.23±2.42 |  | 19.85±3.97 |  | 8.00±2.20 |  | 8.29±2.45 |  | 19.00±3.78 |  | 5.98±1.99 |  | 7.55±2.46 |  | 16.43±4.08 |  | 5.09±1.68 |  |
| Yes | 9.45±2.49 |  | 21.33±4.04 |  | 7.57±2.28 |  | 8.38±2.06 |  | 19.25±4.01 |  | 6.14±1.92 |  | 7.53±2.38 |  | 18.19±4.08 |  | 5.55±1.69 |  |
| Management of community |  | 0.026 |  | 0.738 |  | 0.397 |  | 0.081 |  | 0.006 |  | 0.155 |  | 0.023 |  | 0.581 |  | 0.581 |
| Bad | 7.73±2.89 |  | 21.00±6.00 |  | 7.80±2.18 |  | 8.29±1.44 |  | 20.50±4.38 |  | 6.43±1.65 |  | 6.92±2.43 |  | 16.42±3.90 |  | 16.42±3.90 |  |
| Fair | 9.41±2.03 |  | 21.31±4.11 |  | 8.06±2.31 |  | 7.95±2.08 |  | 18.09±3.86 |  | 5.77±1.97 |  | 7.08±2.22 |  | 17.78±4.05 |  | 17.78±4.05 |  |
| Good | 9.53±2.51 |  | 20.81±3.87 |  | 7.57±2.26 |  | 8.73±2.31 |  | 19.94±3.72 |  | 6.33±1.92 |  | 8.19±2.47 |  | 17.59±4.34 |  | 17.59±4.34 |  |
| Availability of recreational activity |  | 0.687 |  | 0.770 |  | 0.469 |  | 0.708 |  | 0.417 |  | 0.998 |  | 0.038 |  | 0.016 |  | 0.000 |
| No | 9.35±2.27 |  | 20.89±3.92 |  | 7.64±2.27 |  | 8.38±2.13 |  | 19.06±3.84 |  | 6.09±1.97 |  | 7.34±2.34 |  | 17.18±4.12 |  | 5.13±1.47 |  |
| Yes | 9.56±3.22 |  | 21.09±4.67 |  | 7.91±2.25 |  | 8.22±2.39 |  | 19.69±4.36 |  | 6.09±1.82 |  | 8.46±2.52 |  | 19.42±3.88 |  | 6.58±2.15 |  |
| Availability of health education |  | 0.954 |  | 0.063 |  | 0.044 |  | 0.829 |  | 0.051 |  | 0.806 |  | 0.850 |  | 0.064 |  | 0.372 |
| No | 9.39±2.50 |  | 20.75±4.07 |  | 7.58±2.22 |  | 8.37±2.07 |  | 18.82±3.96 |  | 6.07±1.97 |  | 7.52±2.36 |  | 17.27±4.19 |  | 5.32±1.72 |  |
| Yes | 9.41±2.24 |  | 22.24±3.84 |  | 8.48±2.43 |  | 8.29±2.47 |  | 20.16±3.76 |  | 6.16±1.85 |  | 7.63±2.63 |  | 19.00±3.72 |  | 5.67±1.61 |  |

**TABLE 4 |** Multiple linear regression analysis of social support among non-frail participants.

| **Predictor variables** | **Outcome variables** | | | | | | | | | | | |
| --- | --- | --- | --- | --- | --- | --- | --- | --- | --- | --- | --- | --- |
|  | **Overall social support** | | | **Objective support** | | | **Subjective support** | | | **Support utilization** | | |
|  | ***Beta*** | ***t*** | ***p*** | ***Beta*** | ***t*** | ***p*** | ***Beta*** | ***t*** | ***p*** | ***Beta*** | ***t*** | ***p*** |
| (Constant) |  | 10.895 | 0.000 |  | 7.015 | 0.000 |  | 7.563 | 0.000 |  | 9.433 | 0.000 |
| Age (years) (Ref^#^:60-69) |  |  |  |  |  |  |  |  |  |  |  |  |
| 70-79 | -0.151 | -2.551 | 0.011 | -0.227 | -3.720 | 0.000 | -0.138 | -2.312 | 0.022 | 0.026 | 0.396 | 0.693 |
| ≥80 | 0.157 | 2.668 | 0.008 | 0.064 | 1.049 | 0.295 | 0.077 | 1.302 | 0.194 | 0.177 | 2.699 | 0.007 |
| Marital status (Ref: Married) |  |  |  |  |  |  |  |  |  |  |  |  |
| Single^*^ | -0.202 | -3.255 | 0.001 | - | - | - | -0.253 | -3.985 | 0.000 |  |  |  |
| Educational level (Ref: Uneducated) |  |  |  |  |  |  |  |  |  |  |  |  |
| Elementary school | 0.157 | 2.020 | 0.045 | 0.031 | 0.390 | 0.697 | 0.120 | 1.524 | 0.129 | 0.143 | 1.567 | 0.119 |
| Junior high school | 0.228 | 2.736 | 0.007 | 0.112 | 1.314 | 0.190 | 0.234 | 2.847 | 0.005 | 0.052 | 0.535 | 0.593 |
| Senior high school | 0.258 | 3.138 | 0.002 | 0.108 | 1.278 | 0.203 | 0.245 | 3.239 | 0.001 | 0.165 | 1.702 | 0.090 |
| College or above | 0.299 | 3.319 | 0.001 | 0.330 | 3.559 | 0.000 | 0.174 | 2.445 | 0.015 | 0.268 | 2.592 | 0.010 |
| Living alone (Ref: No) |  |  |  |  |  |  |  |  |  |  |  |  |
| Yes | - | - | - | -0.233 | -3.619 | 0.000 | - | - | - | - | - | - |
| The number of children (Ref: ≤1) |  |  |  |  |  |  |  |  |  |  |  |  |
| ≥ 2 | -0.149 | -2.317 | 0.021 | - | - | - | - | - | - | - | - | - |
| Relationship with children (Ref: Poor) |  |  |  |  |  |  |  |  |  |  |  |  |
| Fair | 0.127 | 1.135 | 0.258 | - | - | - | 0.089 | 0.769 | 0.443 | - | - | - |
| Good | 0.368 | 3.274 | 0.001 | - | - | - | 0.331 | 2.805 | 0.005 | - | - | - |
| Management of community (Ref: Bad) |  |  |  |  |  |  |  |  |  |  |  |  |
| Fair | - | - | - | 0.235 | 2.253 | 0.025 | - | - | - | - | - | - |
| Good | - | - | - | 0.234 | 2.217 | 0.028 | - | - | - | - | - | - |
| Model fit | *F*=10.070 | | | *F*=7.763 | | | *F*=8.127 | | | *F*=2.815 | | |
|  | (*p*=0.000) | | | (*p*=0.000) | | | (*p*=0.000) | | | (*p*=0.003) | | |
|  | *R^2^*=0.386 | | | *R^2^*=0.359 | | | *R^2^*=0.353 | | | *R^2^*=0.110 | | |

*^#^*Ref: reference; ^*^Single: including unmarried, divorced, or widowed.

**TABLE 5 |** Multiple linear regression analysis of social support among pre-frail participants.

| **Predictor variables** | **Outcome variables** | | | | | | | | | | | |
| --- | --- | --- | --- | --- | --- | --- | --- | --- | --- | --- | --- | --- |
|  | **Overall social support** | | | **Objective support** | | | **Subjective support** | | | **Support utilization** | | |
|  | ***Beta*** | ***t*** | ***p*** | ***Beta*** | ***t*** | ***p*** | ***Beta*** | ***t*** | ***p*** | ***Beta*** | ***t*** | ***p*** |
| (Constant) |  | 8.931 | 0.000 |  | 10.923 | 0.000 |  | 9.765 | 0.000 |  | 19.037 | 0.000 |
| Marital status (*^#^*Ref: Married) |  |  |  |  |  |  |  |  |  |  |  |  |
| *Single | -0.289 | -3.625 | 0.000 | -0.295 | -4.008 | 0.000 | -0.314 | -3.944 | 0.000 | - | - | - |
| Monthly income ($) (Ref: <300) |  |  |  |  |  |  |  |  |  |  |  |  |
| 300-600 | - | - | - | - | - | - | -0.239 | -2.287 | 0.024 | - | - | - |
| 601-1051 | - | - | - | - | - | - | -0.154 | -1.409 | 0.161 | - | - | - |
| ≥1052 | - | - | - | - | - | - | 0.031 | 0.345 | 0.731 | - | - | - |
| Living alone (Ref: No) |  |  |  |  |  |  |  |  |  |  |  |  |
| Yes | -0.203 | -2.550 | 0.012 | -0.388 | -5.265 | 0.000 | - | - | - | - | - | - |
| Financial support from children (Ref: No) |  |  |  |  |  |  |  |  |  |  |  |  |
| Yes | - | - | - | - | - | - | - | - | - | 0.238 | 3.174 | 0.002 |
| Children’s company (Ref: Rarely) |  |  |  |  |  |  |  |  |  |  |  |  |
| Sometime | - | - | - | - | - | - | 0.043 | 0.473 | 0.637 | - | - | - |
| Often | - | - | - | - | - | - | 0.208 | 2.158 | 0.032 | - | - | - |
| Management of community (Ref: Bad) |  |  |  |  |  |  |  |  |  |  |  |  |
| Fair | - | - | - | - | - | - | - | - | - | - | - | - |
| Good | - | - | - | - | - | - | - | - | - | - | - | - |
| Model fit | *F*=8.412 | | | *F*=46.729 | | | *F*=6.338 | | | *F*=4.993 | | |
|  | (*p*=0.000) | | | (*p*=0.000) | | | (*p*=0.000) | | | (*p*=0.001) | | |
|  | *R^2^*=0.323 | | | *R^2^*=0.360 | | | *R^2^*=0.328 | | | *R^2^*=0.109 | | |

*^#^*Ref: reference; ^*^Single: including unmarried, divorced, or widowed.

**TABLE 6 |** Multiple linear regression analysis of social support among frail participants.

| **Predictor variables** | **Outcome variables** | | | | | | | | | | | |
| --- | --- | --- | --- | --- | --- | --- | --- | --- | --- | --- | --- | --- |
|  | **Overall social support** | | | **Objective support** | | | **Subjective support** | | | **Support utilization** | | |
|  | ***Beta*** | ***t*** | ***p*** | ***Beta*** | ***t*** | ***p*** | ***Beta*** | ***t*** | ***p*** | ***Beta*** | ***t*** | ***p*** |
| (Constant) |  | 7.293 | 0.000 |  | 4.782 | 0.000 |  | 8.046 | 0.000 |  | 4.207 | 0.000 |
| Marital status (^#^Ref: Married) |  |  |  |  |  |  |  |  |  |  |  |  |
| *Single | -0.292 | -3.103 | 0.002 | -0.302 | -3.885 | 0.000 | -0.232 | -2.320 | 0.022 | - | - | - |
| Educational level (Ref: Uneducated) |  |  |  |  |  |  |  |  |  |  |  |  |
| Elementary school | - | - | - | - | - | - | -0.018 | -0.190 | 0.850 | - | - | - |
| Junior high school | - | - | - | - | - | - | 0.095 | 1.053 | 0.294 | - | - | - |
| Senior high school | - | - | - | - | - | - | 0.021 | 0.235 | 0.814 | - | - | - |
| College or above | - | - | - | - | - | - | 0.199 | 2.214 | 0.029 | - | - | - |
| Living alone (Ref: No) |  |  |  |  |  |  |  |  |  |  |  |  |
| Yes | - | - | - | -0.310 | -3.788 | 0.000 | - | - | - | - | - | - |
| Relationship with children (Ref: Poor) |  |  |  |  |  |  |  |  |  |  |  |  |
| Fair | 0.240 | 2.064 | 0.041 | 0.314 | 2.792 | 0.006 | 0.143 | 1.160 | 0.248 | - | - | - |
| Good | 0.383 | 3.061 | 0.003 | 0.315 | 2.542 | 0.012 | 0.317 | 2.382 | 0.019 | - | - | - |
| Management of community (Ref: Bad) |  |  |  |  |  |  |  |  |  |  |  |  |
| Fair | - | - | - | 0.175 | 1.530 | 0.128 | - | - | - | - | - | - |
| Good | - | - | - | 0.259 | 2.263 | 0.025 | - | - | - | - | - | - |
| Availability of recreational activity (Ref: No) |  |  |  |  |  |  |  |  |  |  |  |  |
| Yes | 0.197 | 2.482 | 0.014 | 0.156 | 2.464 | 0.015 | - | - | - | 0.384 | 4.155 | 0.000 |
| Model fit | *F*=7.071 | | | *F*=14.250 | | | *F*=4.942 | | | *F*=2.710 | | |
|  | (*p*=0.000) | | | (*p*=0.000) | | | (*p*=0.000) | | | (*p*=0.006) | | |
|  | *R^2^*=0.432 | | | *R^2^*=0.535 | | | *R^2^*=0.366 | | | *R^2^*=0.163 | | |

^#^Ref: reference; *Single: including unmarried, divorced, or widowed.
